# Immune response in glioma’s microenvironment

**DOI:** 10.1515/iss-2019-0001

**Published:** 2021-01-11

**Authors:** Houminji Chen, Ming Li, Yanwu Guo, Yongsheng Zhong, Zhuoyi He, Yuting Xu, Junjie Zou

**Affiliations:** The Second School of Clinical Medicine, Southern Medical University, Guangzhou, P. R. China; The National Key Clinic Specialty, The Engineering Technology Research Center of Education Ministry of China, Guangdong Provincial Key Laboratory on Brain Function Repair and Regeneration, Department of Neurosurgery, Zhujiang Hospital, Southern Medical University, Guangzhou, P. R. China; Department of Neurosurgery, Henan Provical People’s Hospital, Zhengzhou, P. R. China

**Keywords:** cytokines, glioma, immunoregulatory factors, macrophages, microglia, myeloid-derived suppressor cells, tumor microenvironment

## Abstract

**Objectives:**

Glioma is the most common tumor of the central nervous system. In this review, we outline the immunobiological factors that interact with glioma cells and tumor microenvironment (TME), providing more potential targets for clinical inhibition of glioma development and more directions for glioma treatment.

**Content:**

Recent studies have shown that glioma cells secrete a variety of immune regulatory factors and interact with immune cells such as microglial cells, peripheral macrophages, myeloid-derived suppressor cells (MDSCs), and T lymphocytes in the TME. In particular, microglia plays a key role in promoting glioma growth. Infiltrating immune cells induce local production of cytokines, chemokines and growth factors. Further leads to immune escape of malignant gliomas.

**Summary and Outlook:**

The complex interaction of tumor cells with the TME has largely contributed to tumor heterogeneity and poor prognosis. We review the immunobiological factors, immune cells and current immunotherapy of gliomas, provide experimental evidence for future research and treatment of gliomas.

## Introduction

Gliomas are the most common malignant tumors of the central nervous system. The World Health Organization (WHO) classifies gliomas into the following categories: Low-grade gliomas (LGG, grades I and II) are well-differentiated and grow slowly tumors. While advanced gliomas (HGG, grades III and IV) have insufficient differentiation or degeneration, and strongly infiltrate the brain parenchyma. Grade IV glioma, known as glioblastoma (GBM), is characterized by poor survival, high tumor heterogeneity (inter- and intra-tumor), and lack of effective treatment [1]. GBM patients without any intervention have a median survival of fewer than six months. After standardized comprehensive treatment (surgery combined with radiotherapy and chemotherapy), the median survival time is only 14–15 months [2]. Although much progress has been made in research on gliomas, the treatment results are still not satisfactory. Due to the high heterogeneity of glioma, its immunogenicity can also change frequently [3]. A notable example is that when targeted therapy is used alone, due to the pressure of selection, the previously targeted antigen tends to be low-expressed when the glioma recurs [4], making the treatment of glioma more difficult. This article reviews the immune microenvironment in which gliomas are located. By describing the cells and various cytokines associated with glioma immunity in the microenvironment, the mechanism of immune escape, progression, and invasion of gliomas is explained in general. To provide a theoretical basis for the treatment of glioma immunity.

**Figure 1:**
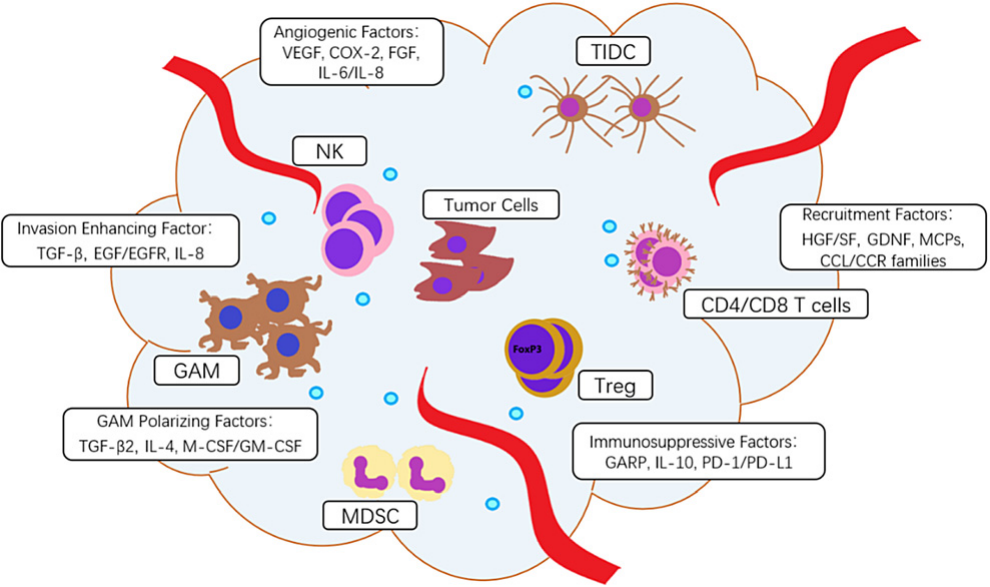
Immune cells and immunoregulatory factors in glioma TME. **(A)** Immune cells include glioma-associated microglia and macrophages (GAMs), T regulatory lymphocytes (Tregs), myeloid-derived suppressor cells (MDSCs), tumor-infiltrating dendritic cells (TIDCs), natural killer (NK), CD4/CD8 T cells. **(B)** Immunoregulatory factors include hepatocyte growth factor (HGF)/scatter factor (SF), glial-derived neurotrophic factor (GDNF), monocyte chemotactic proteins (MCPs), CCL/CCR families, vascular endothelial growth factor (VEGF), cyclooxygenase-2 (COX-2), fibroblast growth factor (FGF), interleukin-6/ interleukin-8 (IL-6/IL-8), glycoprotein A repetition predominant (GARP), interleukin-10 (IL-10), programmed cell death protein 1 (PD1) or its ligand (PD-L1), transforming growth factor β (TGF-β), interleukin-4 (IL-4), macrophage and granulocyte-macrophage colony-stimulating factors (M-CSF, GM-CSF), epidermal growth factor (EGF)/epidermal growth factor receptor (EGFR).

The microenvironment of glioma cells plays an extremely important role in the occurrence and development of tumors. Tumor cells, endothelial cells, immune cells, and a variety of cytokines secreted by the cells together form a glioma tumor microenvironment (TME). The immune cells include macrophages, microglia, regulatory T cells (Tregs), myeloid-derived suppressor cells (MDSCs), T lymphocytes, natural killer cells (NK), dendritic cells, etc. They interact with tumor cells and together play a role in regulating immune effects in the microenvironment. Among them, GAMs play the most important role, and their roles are wide. GAMs can induce immune cells into a pro-inflammatory or anti-inflammatory phenotype, manipulate the immune response, reduce the immune system’s attack on tumor cells, and benefit glioma survival. The structure of ECM is rearranged by GAMs and tumor cells are susceptible to invasion [5], [6]. Various types of cytokines in TME can be divided into chemokines, immunosuppressive factors, angiogenic factors, invasive factors, and important GAM polarizing factors, which promote the transformation of GAMs into the M2 anti-inflammatory and tumor-promoting phenotype (see Figure 1).

## Immune-related cells in glioma microenvironment

Glioma-infiltrated microglia and macrophages, collectively known as glioma-associated microglia and macrophages (GAMs), is the most multi-functional cells group in glioma TAM. This group of cells can take up to 30% of the tumor tissue volume [7]. The number of GAMs infiltrating is positively correlated with tumor grade and poor prognosis [8]. GAMs have strong plasticity and can be polarized into different phenotypes in different microenvironments. Activated GAMs have at least two phenotypes, M1 and M2. M1 and M2 play opposite roles, while M1 plays anti-tumor effects, M2 phenotype has an immunosuppressive effect and secretes TGF-B, IL-6, IL-1B, EGF, and other cytokines to promote the growth, invasion, and expansion of gliomas by stimulating tumor-related blood vessel formation and tumor metastasis. On the other hand, TME also affects the differentiation of GAMs phenotypes. In brain tumors, GAMs are activated by tumor secretion of IL-10 and TGF-B to become an anti-inflammatory and tumor-promoting M2 phenotype. Colony-stimulating factors M-CSF and GM-CSF in glioblastoma can stimulate GAMs to the M2 phenotype. In a glioma mouse model, the use of CSF-1R receptor inhibitors can shrink tumors and prolong the survival time of mice [9]. In addition, GAMs themselves can greatly increase the number of M2 phenotype GAMs by expressing IL-10 and its receptor IL-10R in an autocrine manner [10].

T regulatory lymphocytes (Tregs) are potent immunosuppressive cells. Their immunosuppressive properties are an important cause of glioma immune escape. Tregs do not exist in normal human brain tissue, while a large number of immunosuppressive Tregs are founded in the glioma microenvironment, and the level of Tregs infiltration in glioma is closely related to tumor origin and pathological grade [11]. FoxP3 is an important factor determining Tregs differentiation and maturation. FoxP3 is also the main transcription factor that Tregs regulate the expression of IL-10 and TGF-β [12]. CTL associated antigen-4 (CTLA-4) on Tregs surface, also known as CD152, is generally recognized as an immune checkpoint molecule, which is upregulated in activated CD4+ helper T cells and CD8+ CTLs. Yet, CTLA-4 is constitutively expressed on Tregs and binding to its cognate ligands CD80 and CD86 can induce a lethargy state of matured APCs. Such compromised APCs theoretically lack the ability to activate naive tumor-reactive T-lymphocytes [13]. Tregs can inhibit other immune cells’ functions by secreting cytokines such as IL10 and TGF-β, and induce recruited CD4+T cells in TME to transform into new Tregs, so-called adaptive Tregs [14].

MDSCs are a group of heterogeneous cells, including immature macrophages, granulocytes, DCs, and other myeloid-derived cells at an early stage of differentiation. The infiltration of MDSCs in glioma tissues can exert immunosuppressive effects through a variety of pathways and mediate tumor immune escape. The main manifestations are decreased phagocytosis, increased expression of immunosuppressive molecules IL10, TGF-β [15], and B7H1 [16], inhibited DC differentiation, reduced cytotoxicity of NK cells, and induced T cell apoptosis. CD8+T cells are inhibited by MDSCs through producing reactive oxygen species (ROS) [17] and secreting immune cytokines, as well as inducing Tregs [18]. For CD4+T cells, the expression of PD-1 is induced, which leads to the exhaustion of CD4+ T cells [19]. Elevated GM-CSF levels in the glioma microenvironment can promote MDSCs’ activation, thereby up-regulating the expression of the inhibitory cytokine TGF-β and promoting the formation of an immunosuppressive microenvironment [20]. Various immunosuppressive phenotypes produced by granulocyte-derived MDSCs are related to activation of the STAT3 pathway. Blocking the STAT3 pathway can reduce the gathering of granulocyte-derived MDSCs in the TAM and promote the infiltration of CD4+ and CD8+T cells in tumors [21]. The COX2 pathway can directly promote the generation of systemic MDSCs, and inhibit the infiltration of cytotoxic T lymphocytes (CTL). The use of COX2 inhibitors such as Aspirin will reduce the risk of gliomas [22].

Tumor-infiltrating dendritic cells (TIDCs) can inhibit T cell activation by blocking T cell contact with APCs [23], [24]. In the relationship between DCs and T cells, immature DCs (because of the tumor environment affect) are more likely to cause tumor immune tolerance [25], [26]. Under normal circumstances, DC-derived exosomes promote CTL production while Treg-derived exosomes play a role in inhibition, respectively [27]. However, tumor-infiltrated DCs lose the ability to activate CTL [28]. Researches in DCs are mostly about DC vaccine active immunotherapy for gliomas [29].

Human immune receptor NKp44 expressed by normal natural killer (NK) cells can recognize platelet-derived growth factor (PDGF)-DD produced by tumors, and then trigger NK cells to secrete IFN-γ and TNF-α, thereby inducing tumor cell growth arrest [30]. Compared with other types of tumors, the number of NK cells infiltrating brain tumors is smaller [31]. At the same time, the effectiveness of NK cells in gliomas is also weaker [32]. NK cells cannot complete the physiological processes of cell recognition and killing due to the strong HLA-E expression inhibits NK cells expressing cognate inhibitory killer-cell immunoglobulin-like receptors (KIR) and CD94/NKG2A [33]. Some studies have found that lectin-like transcript 1 (LLT1) expressed on the surface of glioma [34], LLT1 can interact with CD161 on the surface of NK cells [35], and inhibits the cytotoxicity and IFN secretion of NK cells [36].

T cells in TME are affected by chemokines. Among T cell phenotypes, there are more CD4+T cells than CD8+T cells. CD4+T cells often show exhausted markers (expressing Immunoglobulin mucin-3 TIM-3 and death protein PD-1). However, although CD4+T cells are exhausted, they can still secrete IFNγ and continue to promote the migration of T cells to the CNS [37]. It can be observed in murine glioma models that CD8+T cells usually first lose functions such as IL-2 production, high proliferative capacity, and *ex vivo* killing. The ability to secrete tumor necrosis factor is usually lost with the loss of various important functions [38]. CD25(−) is characteristic of disabled CD8+T cells [39]. Immunosuppressive TME inhibits CD8+T cells activation by inducing the expression of high levels of co-suppressing receptors like PD-1/PD-L1 and CTLA-4 [40]. T cell exhaustion has been found in extensive studies of various tumors [41], [42]. The metabolic restrictions imposed by tumors can mediate a low T cell response. PD-1 overexpressed by T cells mediated the upregulation of glucose transporter-1 GLUT1 through the mTOR pathway to make T cells enter a high glucose glycolysis state. In this state, T cells compete with tumor cells for glucose in TME and gradually fail. They cannot maintain the expression of T cell receptor TcR and lose their ability to fight tumors [43]. Clinically used checkpoint blocking antibodies against CTLA-4, PD-1, and PD-L1 can restore glucose concentration in the TME, allowing T cell glycolysis and IFN-γ production. Blocking PD-L1 in tumors can inhibit glycolysis by inhibiting mTOR activity and reducing the expression of glycolytic enzymes, which reflects the role of PD-L1 in tumor glucose utilization [40].

## Immunoregulatory factors

### Recruitment factors

Hepatocyte growth factor (HGF)/scatter factor (SF) is a ligand of transmembrane tyrosine kinase receptor c-Met [44]. Its presence in normal tissue is usually mediated by hypoxia, and is also involved in wound repair [45]. The HGF/SF together with c-Met was found upregulated in glioma tissue, positively correlated with the grade of glioma and poor prognosis [46]. HGF/SF plays a role in the motility and mitosis of gliomas [47], also act as a chemokine for microglial cells and BM-macrophages [44]. The mechanism is revealed as HGF/SF upregulates CXCR4 via NF-κB, which leads to enhanced migration [48].

Glial-derived neurotrophic factor (GDNF) has been identified as a potent neurotrophic factor in a variety of neuronal cell populations and is overexpressed in human gliomas [49]. *In vitro* migration tests showed that GDNF is a strong chemical attractant of microglia but does not affect glioma-induced astrogliosis. GDNF promotes glioma migration and progression in an autocrine manner [50].

Monocyte chemotactic proteins (MCPs) belong to the MCP subgroup of the C–C chemokines family and promote chemotaxis of immune cells [51]. The presence of MCP-1/CCL2 induces microglial proliferation and migration by exclusively binding CCR2 [51]. It was demonstrated several glioma cells mainly express MCP-3 but not MCP-1 [52]. MCP-3 promotes chemotaxis of immune cells by binding to CCR1, CCR2, and CCR3 receptors [53], thus promote the migration of macrophages, monocytes, NKs, T cells, and DCs.

Chemokines and receptors are involved in the development of glioma. CCL5/CCR5 are highly expressed in human glioblastoma and is associated with poor prognosis. CCR5 blockade prevents the M2 microglia phenotype, also significantly reduces microglial migration, which is mediated by inhibition of the AKT pathway [54]. In glioblastoma, increased CCL2 expression is associated with decreased OS. Glioma-derived CCL2 acts on microglia with CCR2 and then produces IL-6 to stimulate the glioma [55]. Tumor cells recruit Tregs and MDSCs through the CCL2 / CCR2 axis to produce local immunosuppression [56]. CX3CL1 expressed by GB cells induces the recruitment of human GAMs through its receptor CX3CR1 and increases the expression of matrix metalloproteases 2, 9, and 14 in GAM, thereby promoting tumor invasion [57]. CXCL12 has been shown to be associated with tumor angiogenesis and tumor cell hypoxia tolerance in an *in vivo* glioma model [58]. Blockade of the CXCL12/CXCR4 axis after irradiation inhibited the recurrence of spontaneous brain tumors in rats [59].

### Angiogenic factors

Glioma cells stimulate microglial cells to increase TGF-B production. It plays a key role in the progression of gliomas by inducing several genes involved in many oncogenic processes such as proliferation, angiogenesis, and invasion [60], [61]. TGF-B activates the Ras protein pathway and MAPK pathway to further mediate the proliferation of high-grade glioma cells and up-regulate the expression of vascular endothelial growth factor (VEGF) and its downstream receptors [62], [63]. It is worth noting that the abundant VEGF caused a decrease in the accumulation of microglia/macrophages in the surrounding environment of blood vessels, and at the same time reduced the release of pro-angiogenic factors (such as VEGF), indicating that there may be a negative feedback mechanism [64]. At the same time, the hypoxia-inducible factor HIF can also induce the expression of VEGF and promote the formation of the tumor vascular system [65].

Cyclooxygenase-2 (COX-2) is an enzyme involved in the production of eicosanoids acid, which plays a major role in GBM angiogenesis. Its effect is achieved by mediating vascular epithelial growth factor VEGF [66]. Fibroblast growth factor (FGF) is also an angiogenesis-inducing factor. FGF can promote the division and chemotaxis of vascular endothelial cells and can participate in the PI3K/AKT signaling pathway to inhibit apoptosis of endothelial cells. Targeted inhibition of COX-2 and FGF expression down-regulates VEGF, thereby slowing the process of angiogenesis [67]. COX2 can be metabolized into prostaglandin E2 (PGE2) [68]. COX2/PGE2 induces the production of Id1 through the EP4-dependent MAPK signaling pathway and the activation of the Egr1 transcription factor. Id1 can increase the resistance of glioblastoma to radiation therapy [69].

Th2-cytokine (IL-6/IL-8) are both considered major regulators of glioma cell growth and invasiveness. STAT-3 is a downstream signal transducer of cytokine signaling and is positively correlated with tumor angiogenesis [70]. After the inhibition of STAT3 is released, IL-6 activates the STAT3 signal cascade, which leads to an increase in the expression level of VEGF in glioma and contributes to tumor vessel formation [71], [72]. IL-8 strongly promotes angiogenesis, and its mechanism of action is to regulate the survival and proliferation of endothelial cells [73].

### Immunosuppressive factors

Glycoprotein A repetition predominant (GARP) is a surface molecule of regulatory T cells and tumor cells. It plays an important role in preventing inflammatory diseases such as allergies and graft-versus-host disease (GvHD). GARP is often hijacked by tumor cells to promote tumorigenesis [74]. Recent research indicates that GARP expression was shown in glioblastoma cell lines, primary glioblastoma tissues, and LGGs. GARP is located on the surface of tumor cells and in the cytoplasm of glioma cells. GARP induces Treg differentiation and M2 phenotype Macrophages and suppresses (tumor antigen-specific) T effector cells, thereby contributing to the immunosuppressive TME of primary brain tumors [75].

GBM patients have elevated levels of immunosuppressive cytokine IL-10 [5]. IL-10 is usually produced when tumor cells are stimulated by M2 macrophages, which in turn can promote the transformation of macrophages to the M2 phenotype. IL-10 can stimulate the transcription factor STAT3 and induce the expression of anti-inflammatory molecules such as TGF-β and FGL2 [76], [77]. At the same time, IL-10 can also inhibit the expression of pro-inflammatory molecules, activate Tregs, inhibit CD8-mediated cytotoxic effects, and silence phagocytosis and antibody expression, which is generally beneficial to tumor survival [78].

Indoleamine 2,3 dioxygenase 1 (IDO1) is a tryptophan catabolism enzyme commonly found in malignant brain tumors, which is related to the downstream kynurenic acid metabolic pathway [79]. Up-regulation of IDO1 expression in glioma patients is associated with decreased overall survival (OS) [80]. The high expression of IDO1 leads to an increase in the level of kynurenic acid. Early studies suggest that the level of kynurenic acid is related to CD4+T cell apoptosis [81]. Current research shows that kynurenic acid may induce the occurrence of Treg through FOXP3, thereby causing tumor immune escape [82], [83].

Arginase is involved in a variety of cellular activities in the glioma microenvironment, including the polarization of tumor-associated glial cells and macrophages [84]. The increase of arginase in blood samples of patients with glioma suggests that it is related to the inhibitory effect of MDSC on tumor immunity [18]. It is known that the arginase produced by MDSC reduces the level of l-arginine that is essential for normal T cell function, leading to T cell dysfunction [85]. In addition, MDSC also uses nitric oxide synthase (NOS) to convert the raw material l-Arginine into nitric oxide that has a wide range of mediating inflammation, angiogenesis, T cell dysfunction and tumor immune escape [86].

The programmed cell death protein 1 (PD1) or its ligand (PD-L1) shows activity in several cancer types [87]. B7-homolog 1 (B7-H1) doesn’t express in normal brain tissue [88]. B7-H1 acts as a ligand of PD1 and inhibits the immune response of TH cells [89]. In addition, B7-H1 can also act as a receptor that transmits anti-apoptotic signals, thereby inhibiting the lysis of cancer cells by cytotoxic CD8 T lymphocytes [90]. Other members of the B7 family, such as B7-H3 and B7-H4, are associated with immune escape in many malignancies and are expected to become new targets for targeted therapy [91], [92], [93]. Fibrinogen-like protein 2 (FGL2) is a secreted factor and is overexpressed in glioblastoma compared to LGGs. FGL2 enhances the immunosuppressive effect of glioma by increasing the expression level of PD1, increasing the M2 phenotype macrophages, and increasing the number of MDSCs and Tregs [94]. At the same time, FGL2 promotes glioma progression by inhibiting CD103+ dendritic cell differentiation [95].

### GAM polarizing factors

Transforming growth factor β (TGF-β) has multiple roles in the process of tumorigenesis. From the occurrence, growth, and invasion of gliomas, TGF-β is involved [96]. TGF-β has three isoforms in mammals, TGF-β1, TGF-β2, and TGF-β3. The three isoforms are roughly similar in structure but distributed in different organs and tissues. Among them, TGF-β2 is abundantly expressed in gliomas and is positively correlated with glioma grades [97]. TGF-β2 works synergistically with prostaglandin E2 to promote polarization by inhibiting the expression of MHC I and MHC class II molecules on the surface of gliomas [98]. TGF-β2 secreted by GAM up-regulates TGF-β receptors of tumor cells and promotes tumor growth [99].

Interleukin-4 (IL-4) is a multifunctional cytokine mainly secreted by Th2 cells, eosinophils, basophils, and stromal cells. IL-4 is known to regulate a variety of immune responses, including the process of T cell and B cell differentiation. It is currently considered to be the best characterizing promoter of M2 polarization in microglia and macrophages [100]. IL-4 promotes microglia M2 polarization through the PI3K pathway and activates the most important downstream transcription factor IRF4. Other members of the IRF family such as IRF7 also participate in a similar process, which is also mediated by IL-4. IL-13 stimulation of microglial cells can polarize the microglia to anti-inflammatory M2 phenotype and repair tissues, playing a role similar to IL-4 [101].

Macrophage and granulocyte-macrophage colony-stimulating factors (M-CSF, GM-CSF) play an important role in the differentiation and development of macrophages. Glioma has a high expression of GM-CSF, and the expression level is negatively correlated with the prognosis of patients. Glioma-derived M-CSF promotes differentiation of macrophages into the M2 phenotype [102]. Although GM-CSF receptor inhibitors cannot reduce the number of infiltrating macrophages, they can weaken their immunosuppressive function [9]. Regulating the number of infiltrating macrophages in gliomas through CSF inhibitors, or changing the immunosuppressive characteristics of infiltrating macrophages is expected to improve the prognosis of glioma patients. GM-CSF has been widely used as an adjuvant in various tumor cell and DC vaccine-related clinical studies [103].

### Invasion enhancing factor

A distinct feature of gliomas is that they infiltrate into the brain parenchyma, and the brain extracellular matrix (ECM) and its regulators play a key role in glioma cell invasion [104]. Fibroblasts found in the cancer microenvironment can exhibit special phenotypes, such as increased expression of α- smooth muscle actin (α-SMA) and fibroblast activating protein (FAP), and increased secretion of ECM proteins, including Fibronectin and type I collagen [105]. During the polarization of GAMs to M2 phenotype, lactadherin, and osteopontin released by tumor cells promote actin filament contraction and microtubule rearrangement, thereby enhancing the M2 phenotype migration ability [106]. GAM-derived TGF-β2 and β1 have been shown to increase matrix metalloproteinase 2 (MMP-2) expression, thereby promoting ECM deposition and promoting glioma invasion [6]. MMP-2 reshapes ECM by degrading type IV and V collagen, promotes tumor neovascularization and helps tumor invasion and expansion [107].

Epidermal growth factor receptor (EGFR) gene amplification is the most common genetic change in primary GBM (about 40%), and high EGFR expression is associated with primary human tumors. The mechanism of glioma cell invasion includes the up-regulation of ECM-degrading proteases and the activation of abnormal signal pathways leading to the degradation of ECM. Epidermal growth factor (EGF) secreted by tumor cells and corresponding immune cells cooperates with up-regulated EGFR to accelerate this process [108]. A mutant form of EGFR, commonly called EGFRvIII or ΔEGFR, is only expressed in tumor cells. EGFRvIII-positive tumors are associated with poor prognosis and short life expectancy. EGFRvIII promotes ECM degradation of gliomas through NF-κB and interleukin 8 pathways while promoting angiogenesis [109]. It is known that EGFR activation promotes MMP-9 expression in different cell types. For example, EGF stimulates ovarian cancer cell migration and MMP-9-dependent invasion [110]. Similar to wild type EGFR, EGFRvIII expression is also closely related to MMP-9 expression. Some scholars speculate that it is regulated by the MAPK/ERK pathway because EGFRvIII is known to activate the extracellular signal-regulated kinase ERK 1/2 in glioma cells, which is a direct regulator of MMP-9 secreted by glioma cells [108].

Interleukin 8 (IL-8) has been shown to be a key regulator of CNS function and development in many CNS0 diseases, including gliomas [111]. Prostaglandin E2 (PGE2) is usually up-regulated in tumor cells, acts on CEBP-β binding sites, and induces demethylation of IL-8 related DNA on CpG islands. At the same time, PGE2 induced H3 acetylation in the IL-8 promoter, which further enhanced its expression [112]. Among different types of tumors, it has been reported that IL-8 promotes tumor invasion through NF-κB [113]. The binding of IL-8 to CXCR2 most effectively triggered the redistribution of VE-cadherin from the junction of endothelial cells. Therefore, vascular permeability is increased, and blood vessels are susceptible to invasion and blood-borne metastasis [114]. Finally, IL-8 participates in cytoskeleton rearrangement, and the IL-8-NF-κB signaling axis promotes F-actin polymerization and mediates epithelial–mesenchymal transition (EMT), promoting the tumor’s growth and invasion [115].

## Immunotherapy of glioma

### Immune checkpoint inhibitor

It is currently believed that the immune checkpoint PD-1 and its ligand PD-L1, as well as CTLA-4, are important factors that inhibit tumor T cell immunity. Anti-PD-1, PD-L1, and CTLA-4 antibodies have shown the potential for tumor regression [116]. However, the prospect of applying PD-1/PD-L1 inhibitors to gliomas is still unclear. In terms of expression level, gliomas have limited expression of PD-1/PD-L1 immune checkpoint [117], which indicates that the efficacy of immune checkpoint inhibitors used alone is limited. In fact, clinical studies using PD-1 inhibitors in recurrent high-grade gliomas have shown a lower response rate [118]. The high expression of CTLA-4 is closely related to poor prognosis [119]. In the mouse glioma model, CTLA-4 inhibitors showed significant efficacy when combined with other immune checkpoint inhibitors [120]. In view of the high heterogeneity of gliomas, a combination of immune checkpoint inhibitors may be a good treatment strategy [121].

### CAR-T cells

Chimeric antigen receptor (CAR)-T cells can specifically recognize tumor cells and target EGFRvIII, IL-13Rα2, and HER2 antigens expressed by glioblastoma. CAT-T cell therapy has been used in clinical trials [122]. Dual-targeting CAR-T cells against EGFR/EGFRvIII showed specific toxicity to glioblastoma in a study [123]. CAR-T cells that double target IL-13Rα2 and HER2 also show good potential for improving the immune escape of glioblastoma [124].

### Vaccine

Similar to the antigen targeted by CAR-T cells, the vaccine target needs to cover tumor-specific antigens such as EGFRvIII and IL-13Rα2 [125]. Due to the high degree of heterogeneity of gliomas and the selective pressure brought by immunotherapy, gliomas that relapse after immunotherapy often lose the expression of specific antigens [4]. Combining vaccines against multiple antigens may avoid this shortcoming, but there is not enough evidence to support this view [122].

## Conclusions

The dynamic interaction between glioma cells and their TME plays a pivotal role in the growth and development of tumors, and it also brings major challenges to treatment. TME of gliomas not only inhibit the anti-tumor immune response but also significantly promote the occurrence and development of gliomas. Among various cell types, GAMs play the most critical role. The key problem to be solved is the dynamic observation of changes in the immunological characteristics of GAMs during the development of gliomas, including migration and accumulation in the local area of ​​gliomas Cellular and molecular mechanisms that exert inhibitory effects. In addition, studying immune cells such as MDSC, DC, and Treg and their secreted cytokines, and carrying out targeted immunotherapy may combat gliomas, especially GBM, and can supplement surgery and chemotherapy. Through an in-depth discussion of the above-mentioned series of issues, not only can we make several discoveries in the theoretical study of the local regional immune characteristics of gliomas, but also provide experimental evidence for the comprehensive diagnosis and treatment of gliomas’ immune microenvironment. The limited knowledge gained from the research will provide inspiration for future research and treatment of gliomas.
