# Supplementary material for: Immune response in glioma’s microenvironment
Source: Innov Surg Sci. 2021 Jan 11;5(3-4):115–25. doi: 10.1515/iss-2019-0001 (PMC7810204; doi:10.1515/iss-2019-0001)
Supplement: Supplementary file 1 [file iss-05-20190001-s001.pdf]

Reviewer Assessment

H. Chen, M.Li and Y Guo: Immune Response in Glioma's Microenvironment

Reviewers' Comments to Original Submission

Reviewer 1: anonymous

Date received: 14-May-2020
Reviewer recommendation: **Return to author for major modifications**
Reviewer overall scoring: **Medium**

Assessment Form scores: 5 = High/Yes; 3 = Medium/Adequate; 1 = Low

Is the subject area appropriate for the journal					
Does the title clearly reflect the paper's content?				2	
Does the abstract clearly reflect the paper's content		4			
Do the keywords clearly reflect the paper's content?	5				
Does the introduction present the problem clearly?	5				
Are the results/ conclusions justified?			3		
How comprehensive and up-to-date is the subject matter presented?			3		
How adequate is the data presentation?			3		
Are units and terminology used correctly?		4			
Is the number of cases adequate?		4			
Are the experimental methods/ clinical studies adequate?			3		
Is the length appropriate in relation to the content?			3		
Does the reader get new insights from the article?		4			
Please rate the practical significance.	5				
Please rate the accuracy of methods.			3		
Please rate the statistical evaluation and quality control.	5				
Please rate the appropriateness of the figures and tables.	5				
Please rate the appropriateness of the references.				2	
Please evaluate the writing style and use of language.				2	
Please judge the overall scientific quality of the manuscript.			3		
Are the methods used worthy of reproduction in greater deal?	No				
Would you be willing to review a revision of this manuscript?	No				

Comments to author: Major critics:
Title does not properly reflect the topic of the review. It is recommended to alter the title to "Immunosuppressive mechanisms of glioma tumor microenvironment"

In general the manuscript is suitable for publication once the major critical points raised by the reviewer has been addressed (see below)

It is advised to implement a separate chapter prior the discussion sections which describes recent and novel approaches or studies to improve immunotherapy of glioma

There are several statements of the authors that are not in line with common knowledge or are not supported by accompanying citations
In some cases, the authors refer to studies using mouse models, which due to species-specific differences are misleading
The manuscript appears immature, containing numerous typing errors and incomplete sentences. Style must be improved by an native speaker....
Examples are
....T cells in TME are affected by chemokines. Among T cell ...phnotypes..., CD4+T cells often show exhausted ....makers...
....However, tumor-infiltrated DCs lose the ability to mediate CTL[19]....
... B7-homolog 1 (B7-H1) ....dont expresse... on normal brain tissue[68].....

Page 3, starting with second paragraph
“Cytotoxic T lymphocyte associated antigen4 (CTLA-4) on Tregs surface, also known as CD152, is an inhibitory molecule that can inhibit the activation of effector T cells by binding to CD80 and CD86 on the surface of antigen-presenting cells [11]. Tregs can inhibit other immune cells’ functions by secreting cytokines such as IL10 and TGF-β, and induce recruited T cells in TME to transform into new Tregs[12].”
...is somewhat misleading and cites irrelevant literature.
Correct to;
“Cytotoxic T lymphocyte associated antigen4 (CTLA-4) on Tregs surface, also known as CD152, is generally recognized as immune checkpoint molecule, which is upregulated in activated CD4+ helper T cells and CD8+ cytotoxic T lymphocytes. Yet, CTLA-4 is constitutively expressed on Tregs and binding to its cognate ligands CD80 and CD86 can induce a lethargy state of matured APCs. Such compromised APCs theoretically lack the ability to activate naïve tumor-reactive T-lymphocytes (Chen J, Ganguly A, Mucsi AD, Meng J, Yan J, Detampel P, et al. (February 2017). "Strong adhesion by regulatory T cells induces dendritic cell cytoskeletal polarization and contact-dependent lethargy". The Journal of Experimental Medicine. 214 (2): 327–338.) Tregs can inhibit other immune cells’ functions by secreting cytokines such as IL10 and TGF-β, and induce recruited CD4+ T cells in TME to transform into new Tregs, so called adaptive Tregs (M. R. Walker, D. J. Kasprowicz, V. H. Gersuk et al.,Induction of FoxP3 and acquisition of T regulatory activity by stimulated human CD4+CD25− T cells,” Journal of Clinical Investigation, vol. 112, no. 9, pp. 1437–1443, 2003).

Citation]13] is not covering all aspects listed in the following paragraph ...”The infiltration of MDSCs in glioma tissues can exert immunosuppressive effects through a variety of pathways and mediate tumor immune escape. Which decreased phagocytosis, increased expression of immunosuppressive molecules, such as IL10, TGF-β, and B7H1, inhibited DC differentiation, reduced cytotoxicity of NK cells, and induced T cell apoptosis. CD8+T cells are inhibited by MDSCs through producing reactive oxygen species (ROS) and secreting immune cytokines, as well as inducing Tregs[13]....
please include citations demonstrating decreased phagocytosis, secretion of IL-10, TGF-beta, increased surface expression of B7-H1 by MDSCs (which is PD1-L!!!!----->better included in chapter “immunosuppressive factors”) and production of ROS by MDSCs. In [13] increased arginase levels in GBM patients are described, which can be produced by MDSCs (recommended to include in chapter “Immunosuppressive Factors”)

Elevated GM-CSF levels in the glioma microenvironment can promote MDSCs activation of STAT6 and STAT1 signaling pathways, Up-regulating the expression of the inhibitory cytokine TGF-β and promoting the formation of an immunosuppressive microenvironment.
Citation is missing!

On the other hand, the COX2 pathway can directly promote the generation of systemic MDSCs, and inhibit the infiltration of cytotoxic T lymphocytes (CLT). COX2 inhibitors will reduce the risk of glioma[16].

This paragraph better fits into the chapter “Immunosuppressive Factors”. Yet, for the interested readership, it is worthwhile to explain action of COX2 and effects of produced PGE2….

Tumor-infiltrating dendritic cells (TIDCs) can suppress T cell immunity and participate in the progression of glioma[17].
[17] is irrelevant citation! Include appropriate citation!

TIDCs upregulate PD-1 (don´t think that this is correct, include citation for this), inhibits T cell activation by blocking T cell contact with APCs. In the relationship between DCs and T cells, immature DCs (confusing! Immature DCs is the same as TIDCs?) are more likely to cause tumor immune tolerance[18]. Under normal circumstances, DC-derived exosomes promote CTL production while Treg-derived exosomes play role in inhibition, respectively. However, tumor-infiltrated DCs lose the ability to mediate CTL[19] (what do the authors mean with “mediate CTLs????).
So far, the research focus about DCs are DC vaccine. The DC vaccine has been proved to be feasible and effective in the Phase I / II clinical trials for GBM[20,21].
[20] and [21] are irrelevant citations and contain only pre-clinical data using mouse models! Include appropriate citation for clinical trials or skip this section!

Page 4, starting first paragraph
Although increasing NK cells in the blood and TME of glioma patients, the function of NK cells was inhibited, and the impaired function was more pronounced in high-grade glioma[23]
[23] is irrelevant citation! Include appropriate citation demonstrating infiltrating NK cells in glioma specimens using IHC or flow cytometry!

NK cells cannot complete the physiological processes of cell recognition and killing due to the blocking of tumor HLA1 molecules and killer-cell immunoglobulin-like receptors (KIR)[18].
The sentence is somewhat misleading. Strong HLA class I expression on glioma cells and strong HLA-E expression inhibits NK cells expressing cognate inhibitory KIR and CD94/NKG2A!

Some studies have found that lectin-like transcript 1 (LLT1) expressed on the surface of glioma [citation is missing]. LLT1 can interact with CD161 on the surface of NK cells[24], and inhibits the cytotoxicity and IFN secretion of NK cells[25]. One study show that the expression level of NKG2A, an inhibitory receptor of NK cells infiltrated in CNS, which increased, on the other hand, a large amount of chemokine CCL2 could be produced, causing monocytes to aggregate in the tumor[26] (confusing! Citation [26] describes an autoimmune setting. It is recommended to skip this paragraph). Since to date there are no convincing studies published, which revealed infiltration of NK cells into glioma the authors should consider to modify or skip this paragraph…

Page 4, starting second paragraph
T cells in TME are affected by chemokines. Among T cell …phnotypes…, CD4+T cells often show exhausted ….makers… (expressing Immunoglobulin mucin-3 TIM-3 and death protein PD-1). However, although CD4+T cells exhausted, they can still secrete IFN-γ and continue to promote the migration of T cells to the CNS[28]. CD8+T cells in glioma usually first lose functions such as IL-2 production, high proliferative capacity, and vitro killing. The ability to secrete TNF is usually lost with the loss of various important functions[29]
(citation irrelevant, since [29] is reviewing mouse T cell exhaustion. Note, IL-2 is not produced by human CD8+ cytototoxic T-lymphocytes!).
CD25 negative (style! Consider revising!) is characteristic of disabled CD8+ T cells. Immunosuppressive TME inhibits CD8+T cells activation by inducing the expression of high levels of co-suppressing receptors (which? Please mention)[30].

Page 5, fourth paragraph
Th2-cytokine (IL-6 / IL-8 (IL-2 is indeed a TH2 cytokine, yet IL-8 is a chemokine (synonym; CXCL8!)) is considered (both or in combination …?) a major regulator which can work on cell growth and invasiveness. STAT-3 is a downstream signal transducer of cytokine signaling and is positively correlated with tumor angiogenesis[58].In glioma models, the inhibitory SOCS3 signal of endothelial cells down-regulated (confusing! Please clarify signaling pathway etc.). After the inhibition of STAT3 is released, IL-6 activates the STAT3 signal cascade, which leads to an increase VEGF level in glioma and contributes

to tumor vessel formation[59,60].IL-8 strongly promotes angiogenesis, which regulate the survival and proliferation of endothelial cells[61].

Page 5, Immunosuppressive factors
It is recommended to include and explain further immunosuppressive factors such as IDO, arginase, and nitric oxide synthetase.

Reviewer 2: Tateishi, Kensuke

Date received: 27-May-2020
Reviewer recommendation: **Return to author for minor modifications**
Reviewer overall scoring: **High**

Assessment Form scores: 5 = High/Yes; 3 = Medium/Adequate; 1 = Low

Is the subject area appropriate for the journal					
Does the title clearly reflect the paper's content?	5				
Does the abstract clearly reflect the paper's content		4			
Do the keywords clearly reflect the paper's content?		4			
Does the introduction present the problem clearly?	5				
Are the results/ conclusions justified?	5				
How comprehensive and up-to-date is the subject matter presented?	5				
How adequate is the data presentation?		4			
Are units and terminology used correctly?		4			
Is the number of cases adequate?		4			
Are the experimental methods/ clinical studies adequate?		4			
Is the length appropriate in relation to the content?			3		
Does the reader get new insights from the article?		4			
Please rate the practical significance.	5				
Please rate the accuracy of methods.		4			
Please rate the statistical evaluation and quality control.		4			
Please rate the appropriateness of the figures and tables.		4			
Please rate the appropriateness of the references.					
Please evaluate the writing style and use of language.			3		
Please judge the overall scientific quality of the manuscript.	5				
Are the methods used worthy of reproduction in greater deal?					
Would you be willing to review a revision of this manuscript?	Yes				

Comments to author: Thank you for the opportunity to review this manuscript about immune response in microenvironment. This review is well written and summarized broad range of immunoregulatory mechanism. In addition to EGFR amplification and EGFR variant III, another few explanation is desired how genetic alterations frequently observed in glioblastoma promote immune reaction.

Authors’ Response to Reviewer Comments

Date received: 09-Sep-2020

Response to reviewer 1

1. Title does not properly reflect the topic of the review. It is recommended to alter the title to “Immunosuppressive mechanisms of glioma tumor microenvironment”.

Author reply: We have revised the title.

Author action: The title has been altered to “Immunosuppressive mechanisms of glioma tumor microenvironment”.

2. It is advised to implement a separate chapter prior the discussion sections which describes recent and novel approaches or studies to improve immunotherapy of glioma.

Author reply: We added a separate chapter “IMMUNOTHERAPY OF GLIOMA”.

Author action: “IMMUNOTHERAPY OF GLIOMA”, the newly added chapter, reviews the recent advances of glioma immunotherapy from aspects of immune checkpoint inhibitor, CAR-T cell, and vaccine, is now located before the conclusion.

3. The manuscript appears immature, containing numerous typing errors and incomplete sentences.

Author reply: We have improved the styles through the whole manuscript by helping hands from a few researchers.

Author action: We improved the expression of specific paragraphs in the manuscript based on the comments of the reviewers, and corrected many minor errors through the full text.

4. Citation [13] is not covering all aspects listed in the following paragraph...please include citations demonstrating decreased phagocytosis, secretion of IL-10, TGF-beta, increased surface expression of B7-H1 by MDSCs.

Author reply: We have supplemented and improved the references based on the reviewers’ comments.

Author action: We have added appropriate references for each MDSCs’ performance mentioned.

Citation of “Elevated GM-CSF levels in the glioma microenvironment can promote MDSCs activation of STAT6 and STAT1 signaling pathways, Up-regulating the expression of the inhibitory cytokine TGF-β and promoting the formation of an immunosuppressive microenvironment.” is missing.

Author reply: We have supplemented and improved the references based on the reviewers’ comments.

Author action: We have added appropriate references and deleted descriptions of unclear signal pathways to avoid misleading readers.

5. “On the other hand, the COX2 pathway can directly promote the generation of systemic MDSCs, and inhibit the infiltration of cytotoxic T lymphocytes (CLT). COX2 inhibitors will reduce the risk of glioma[16]. ”
This paragraph better fits into the chapter “Immunosuppressive Factors”. Yet, for the interested readership, it is worthwhile to explain action of COX2 and effects of produced PGE2.

Author reply: Based on the comments of the reviewers, we have improved the description of COX2 synthesis of PGE2 and supplemented the references.

Author action: Based on the comments of the reviewers, in chapter ANGIOGENIC FACTORS, we have expanded the description of COX2 and briefly reviewed the aspects of COX2/PGE2 promoting tumor resistance.

6. [17] is irrelevant citation!

Author reply: We corrected the text and citation.

Author action: We deleted the first sentence of this paragraph to avoid misleading, and revised the relevant narrative that followed and added relevant references.

7. “immature DCs are more likely to cause tumor immune tolerance [18]”. Immature DCs is the same as TIDCs?

Author reply: The two are not exactly the same, but when DCs are generated in the tumor microenvironment, it will become difficult to mature because of the effect of the tumor. They will present tumor Ags to T cells in a tolerogenic manner and will induce T cell tolerance rather than active anti-tumor immune response. (Strioga M, Schijns V, Powell DJ Jr, Pasukoniene V, Dobrovolskiene N, Michalek J. Dendritic cells and their role in tumor immunosurveillance. Innate Immun. 2013;19(1):98-111.)

Author action: We have revised this sentence with a clearer statement and attached relevant references.

8. “However, tumor-infiltrated DCs lose the ability to mediate CTL[19]” What do the authors mean with “mediate” CTLs?

Author reply: We mean “upregulate” or “activate” CTLs.

Author action: We revised the specific vocabulary based on the reviewers’ comments to avoid sentence ambiguity. Relevant references were added.

[20] and [21] are irrelevant citations and contain only pre-clinical data using mouse models! Include appropriate citation for clinical trials or skip this section.

Author reply: We corrected the citation into appropriate ones. Also, we reviewed this section in a separate chapter.

Author action: We deleted the last sentence of this paragraph, and corrected the citation.

9. [23] is irrelevant citation! Include appropriate citation demonstrating infiltrating NK cells in glioma specimens using IHC or flow cytometry.

Author reply: Regarding the number of NK cell infiltration in gliomas, defining NK cells based on different markers seems to lead to the opposite conclusion. We revised the entire sentence to avoid misleading.

Author action: We revised the entire sentence, and updated the citation.

10. “NK cells cannot complete the physiological processes of cell recognition and killing due to the blocking of tumor HLA1 molecules and killer-cell immunoglobulin-like receptors (KIR) [18]. ”
The sentence is somewhat misleading. Strong HLA class I expression on glioma cells and strong HLA-E expression inhibits NK cells expressing cognate inhibitory KIR and CD94/NKG2A!

Author reply: We are very grateful to the reviewers for their professional suggestions, and this sentence have been revised based on the reviewers' comments.

Author action: We revised the misleading part of the sentence with related citation.

11. Some studies have found that lectin-like transcript 1 (LLT1) expressed on the surface of glioma [citation is missing].

Author reply: We added the appropriate citation.

Author action: We added the appropriate citation.

12. One study shows that the expression level of NKG2A, an inhibitory receptor of NK cells infiltrated in CNS, which increased, on the other hand, a large amount of chemokine CCL2 could be produced, causing monocytes to aggregate in the tumor [26] (confusing! Citation [26] describes an autoimmune setting. It is recommended to skip this paragraph). Since to date there are no convincing studies published, which revealed infiltration of NK cells into glioma the authors should consider to modify or skip this paragraph.

Author reply: Since the result is not clear and the content of this paragraph does not meet the subject of this chapter, we have deleted this paragraph in accordance with the comments of the reviewers.

Author action: The last two sentences of this paragraph have been skipped.

13. CD8+T cells in glioma usually first lose functions such as IL-2 production, high proliferative capacity, and vitro killing. The ability to secrete TNF is usually lost with the loss of various important functions[29] (citation irrelevant, since [29] is reviewing mouse T cell exhaustion. Note, IL-2 is not produced by human CD8+ cytotototoxic T-lymphocytes!).

Author reply: Based on the reviewers' comments, we have added a description of the glioma model species here to avoid misleading readers.

Author action: We have added a description of the murine glioma model in this sentence.

14. CD25 negative (style! Consider revising!) is characteristic of disabled CD8+ T cells. Immunosuppressive TME inhibits CD8+T cells activation by inducing the expression of high levels of co-suppressing receptors (which? Please mention) [30].

Author reply: We have corrected the style of CD25(-) in the manuscript. Also, we listed the co-suppressing receptors.

Author action: We have corrected the style of CD25(-) in the manuscript. Also, we listed the co-suppressing receptors.

15. Th2-cytokine (IL-6 / IL-8) is considered (both or in combination?)

Author reply: We added details to the sentence to clarify the meaning.

Author action: "Th2-cytokine (IL-6 / IL-8) are both considered major regulators..."

16. In glioma models, the inhibitory SOCS3 signal of endothelial cells down-regulated (confusing! Please clarify signaling pathway etc.)

Author reply: To avoid misleading, we deleted this sentence.

Author action: To avoid misleading, we deleted this sentence.

17. It is recommended to include and explain further immunosuppressive factors such as IDO, arginase, and nitric oxide synthetase.

Author reply: Based on the enlightening suggestions of the reviewers, we briefly reviewed IDO, arginase, and nitric oxide synthetase.

Author action: We have added 2 paragraphs in the chapter IMMUNOSUPPRESSIVE FACTORS to briefly review IDO, arginase, and NOS.

Response to reviewer 2

In addition to EGFR amplification and EGFR variant III, another few explanation is desired how genetic alterations frequently observed in glioblastoma promote immune reaction.

Author reply: Based on the reviewer's suggestion, we added a description of the relationship between the mutation of glioma and its immunity.

Author action: We have added a description of glioma heterogeneity and antigenic changes in Chapter INTRODUCTION.

Reviewers' Comments to Revised Submission

Reviewer 2: Tateishi, Kensuke

Date received: 02-Oct-2020
Reviewer recommendation: **Accept in present form**
Reviewer overall scoring: **Excellent**

Assessment Form scores: 5 = High/Yes; 3 = Medium/Adequate; 1 = Low

Is the subject area appropriate for the journal	5				
Does the title clearly reflect the paper's content?	5				
Does the abstract clearly reflect the paper's content		4			
Do the keywords clearly reflect the paper's content?		4			
Does the introduction present the problem clearly?		4			
Are the results/ conclusions justified?	5				
How comprehensive and up-to-date is the subject matter presented?	5				
How adequate is the data presentation?	5				
Are units and terminology used correctly?	5				
Is the number of cases adequate?	5				
Are the experimental methods/ clinical studies adequate?		4			
Is the length appropriate in relation to the content?			3		

Does the reader get new insights from the article?		4			
Please rate the practical significance.		4			
Please rate the accuracy of methods.		4			
Please rate the statistical evaluation and quality control.					
Please rate the appropriateness of the figures and tables.		4			
Please rate the appropriateness of the references.		4			
Please evaluate the writing style and use of language.		4			
Please judge the overall scientific quality of the manuscript.	5				
Are the methods used worthy of reproduction in greater deal?	Yes				
Would you be willing to review a revision of this manuscript?	Yes				

Comments to author: I have no additional comment in this revised manuscript.

Comments by the Editor-in-Chief to Revised Submission

Since the second reviewer rated the revised manuscript "5", I feel that we should accept the revised manuscript, although reviewer 1 wants another revision. Since he is not willing to re-review the manuscript, I tend to accept the paper in this revised version.

Comments by the Editorial Office to the Editor-in-Chief Decision

The pending comments by reviewer 1 were received after the decision was committed. Since all initial comments were addressed by the others in the revision, the decision was finally approved.
The delayed reviewer report stated:

Reviewer 1: anonymous

Date received: 21-Oct-2020
Reviewer recommendation: **Return to author for major modifications**
Reviewer overall scoring: **Low**

Assessment Form scores: 5 = High/Yes; 3 = Medium/Adequate; 1 = Low

Is the subject area appropriate for the journal		4			
Does the title clearly reflect the paper's content?	5				
Does the abstract clearly reflect the paper's content	5				
Do the keywords clearly reflect the paper's content?	5				
Does the introduction present the problem clearly?	5				
Are the results/ conclusions justified?		4			
How comprehensive and up-to-date is the subject matter presented?			3		
How adequate is the data presentation?			3		
Are units and terminology used correctly?				2	

Is the number of cases adequate?		4			
Are the experimental methods/ clinical studies adequate?					
Is the length appropriate in relation to the content?		4			
Does the reader get new insights from the article?		4			
Please rate the practical significance.		4			
Please rate the accuracy of methods.			3		
Please rate the statistical evaluation and quality control.					
Please rate the appropriateness of the figures and tables.					
Please rate the appropriateness of the references.			3		
Please evaluate the writing style and use of language.			3		
Please judge the overall scientific quality of the manuscript.			3		
Are the methods used worthy of reproduction in greater deal?	No				
Would you be willing to review a revision of this manuscript?	No				

Comments to author: In the revised manuscript, the authors partially address my concerns and critics. The manuscript still needs improvement in style and several new citations often do not support or reflect the given statements of the review. In addition, it is advised to avoid citation of reviews and when original research articles are used, it is recommended to give a brief intro whether the results were obtained in human or mouse models as well as referring to the tumor entity investigated. Therefore, it is strongly advised to re-review the cited literature in detail and to consider consulting an immunologist. When accomplishing this task, the manuscript might be suitable for publication.

Some examples leading to my major concern:
Page 10 second paragraph: ref 13 describes reorganization of DC cytoskeleton upon ligation with Tregs, which attenuates DC activity. In the context of the manuscript, this ref. should be substituted with "Kowalczyk A. et al., Cell-extrinsic CTLA4-mediated regulation of dendritic cell maturation depends on STAT3. Eur. J. Immunol. 2014. 44: 1143–1155". This ref. shows downregulation of costimulatory CD80 and CD86 molecules in murine and humane DCs after engagement with CTLA-4-Fc and Tregs.

Page 11 second paragraph: The authors stated that "Tumor infiltrating dendritic cells (TIDCs) can inhibit T cell activation by blocking T cell contact with APCs. [23, 24]" What is meant with blocking T cell contact? Yet, citation 23 identifies TIDCs with poor APC function in human non-small lung cancer, which are potentially still capable of migrating to sentinel lymph nodes. This might result in immunosuppressive effects on naïve tumor reactive T cells….Furthermore, ref. 24 describes that TIGIT, which is expressed on regulatory, memory and activated T cells is a inhibitory receptor of T cells and when ligated to PVR/CD155 on DCs results in decreased IL-12 expression but increased secretion of immunosuppressive IL-10 by DCs. It is recommended to revise this paragraph accordingly.

Page 11 third paragraph: correct to: strong classical HLA- and non-classical HLA-E expression of glioma cells inhibits NK cells expressing cognate inhibitory killer-cell immunoglobulin-like receptors (KIRs) and CD94/NKG2A.

Page 11, third paragraph: "CD8+ T cells usually first lose functions such as IL-2 production", CTLs are commonly not considered as a relevant source for IL-2 (!). In addition, here, it is not recommended to refer to a broad review (ref. [38]) …."Immunosuppressive TME inhibits CD8+ T cells activation by inducing the expression of high levels of co-suppressing receptors like PD-1/PD-L1 and CTLA-4" (???). Sentence is confusing. There is much evidence that tumor cells counteract the attack of immune effector cells by upregulation of inhibitory ligands of the B7 family (i.e. B7-H1/PD-L1) which then bind and inhibit T cells (also when employing CAR-T cells!). Please, revise accordingly.

Minor (examples):

Note: Synonym for B7-H1 is PD-1L! Use one synonym throughout the whole text... Page: Tregs are found instead "Tregs are founded"
Page 11, 4th paragraph: use the term exhaustion marker instead of "exhausted markers"
Page 16 "immune Checkpoint Inhibitor": "In terms of expression levels, gliomas have limited expression of PD-L1/PD-1 (PD-1 expression is on the site of T cells!)) immune checkpoint, (style!! consider consulting of native speaker).
